# Repeatability and Validity of MNREAD Test in Children With Vision Impairment

**DOI:** 10.1167/tvst.9.13.25

**Published:** 2020-12-16

**Authors:** Dawn K. DeCarlo, Liyan Gao, Gerald McGwin, Cynthia Owsley, MiYoung Kwon

**Affiliations:** 1 Department of Ophthalmology and Visual Sciences, School of Medicine, University of Alabama at Birmingham, Birmingham, AL, USA; 2 Department of Optometry, School of Optometry, University of Alabama at Birmingham, Birmingham, AL, USA; 3 Department of Epidemiology, School of Public Health, University of Alabama at Birmingham, Birmingham, AL, USA; 4 Department of Surgery, School of Medicine, University of Alabama at Birmingham, Birmingham, AL, USA

**Keywords:** low vision, vision impairment, pediatric, reading, MNREAD, children

## Abstract

**Purpose:**

To evaluate the test–retest reliability and validity of the MNREAD test for use in children with vision impairment (VI) and to compare their performance on the test to that of normally sighted children.

**Methods:**

Children with VI (*n* = 62) and without VI (*n* = 40) were administered the MNREAD test and the Basic Reading Inventory (BRI) on two study visits, 1 to 3 weeks apart. The maximum reading rate, critical print size, and reading acuity were determined for the MNREAD test, and test–retest reliability was evaluated. The reading rate for the MNREAD test was compared to the BRI results.

**Results:**

Strong correlations between visits were found for all MNREAD parameters (0.68–0.99). Older, but not younger, children with VI read significantly more slowly on both the MNREAD and the BRI than children with normal vision (*P* < 0.05). Reading rates between the two tests were strongly correlated (*r* = 0.88). For the MNREAD test, the reading rate increased 4.4 words per minute (wpm) per year for VI and 10.6 wpm/y for those with normal vision. For the BRI, the reading rate increased by 5.9 wpm/y for VI and 9.7 wpm/y for those with normal vision. Poorer visual acuity was associated with slower reading rates on the MNREAD test but not on the BRI, as the MNREAD relies largely on visual factors but the BRI also relies on linguistic and grammar skills.

**Conclusions:**

The MNREAD test are reliable and valid for use in children with vision impairment.

**Translational Relevance:**

The MNREAD test can be utilized by clinicians, as they are a quick, easy-to-administer method for evaluating reading vision in children with VI.

## Introduction

Much of what we know about the visual requirements for reading with normal or impaired vision comes from the work of Legge and colleagues.^1^ In adults, we know that the integrity of the central visual field accounts for the largest portion of the variance in reading speed.^2^ However, most of the adults with vision impairment in those studies learned to read before they developed vision impairment. The majority of children with vision impairment have enough sight to read visually and do not learn to read Braille.^3^^,^^4^ An extensive literature search revealed that the fastest mean maximum oral reading rate in a study of children with vision impairment was 147 ± 61 words per minute (wpm).^5^ The reading rate in the study by Lovie-Kitchin et al.^5^ increased by 9.9 wpm per year of age, in contrast to the normative data from Carver's study,^6^ in which the reading rate of children with normal vision increased by 14 wpm per year of age. Several additional studies support the finding of decreased reading rates among children with vision impairment.^7^^–^^11^ Because reading rates will vary depending on many factors, including age and grade of child, text size, difficulty, length, mode of presentation, type of reading (oral vs. silent), and skimming versus reading each sentence for comprehension, there is no gold-standard “normal” reading rate for children or adults. Studies using a normally sighted control group provide the best comparisons, as the testing situations are the same.

The MNREAD test are commercially available reading speed and acuity tests that are increasingly being used to measure outcomes after medical treatment for eye diseases such as diabetic retinopathy,^12^ retinal vein occlusion,^13^ and macular hole or pucker^14^ or to evaluate medical devices such as multifocal intraocular lens implants.^15^ The MNREAD test have also been used to determine the effects of vision rehabilitation in adults.^16^^,^^17^ Despite the benefits, use of the MNREAD test in children has been limited,^11^^,^^18^^,^^19^ and the repeatability and validity of the English version have yet to be studied in children with low vision. The MNREAD test use sentences of 10 standard word length (60 characters) to determine reading speed across print sizes that decrease logarithmically, ranging from 8M (11.6 mm *x*-height) to 0.13M (0.19 mm *x*-height). This corresponds to a range from 20/6 (–0.5 logMAR) to 20/400 (1.3 logMAR) when tested at 40 cm. Testing with the MNREAD test yields three reading performance measures: maximum oral reading rate (MRR), reading acuity (RA, smallest print size read), and critical print size (CPS, smallest print size read at the maximum reading rate). More recently, a reading accessibility index has also been developed to provide a single measure reflecting an individual's ability to access print.^20^ The purpose of our study was to investigate the test–retest reliability, as well as the validity of the MNREAD test for children with and without vision impairment.

## Methods

This study was approved by the University of Alabama at Birmingham Institutional Review Board for human use and adhered to the tenets of the Declaration of Helsinki. A parent or guardian provided written informed consent. Children 14 years of age and older also provided written informed consent, whereas younger children provided written assent.

### Participants

Children with vision impairment (VI) not correctable with glasses or contact lenses were recruited for participation from the UAB Center for Low Vision Rehabilitation. Children with normal vision were recruited through flyers placed in the Center's waiting room. Children in grades 1 to 12 were invited to participate. Inclusion criteria for children with VI were bilateral VI of organic etiology and best-corrected visual acuity in the better eye between 0.3 and 1.6 logMAR (20/40 to 20/800). Inclusion criteria for children with normal sight were best-corrected visual acuity in each eye of at least 0.1 logMAR (20/25 or better) and refractive error between +4 diopter (D) and –4 D with no more than 1.5 D astigmatism or 0.75 D anisometropia. Exclusion criteria for both groups included diagnosis of a reading disability, total standard score on the Slosson Intelligence test of ≤85, or the inability to read at a third-grade independent level on the word reading test of the Basic Reading Inventory (BRI).^21^ This reading level was chosen because the MNREAD sentences are comprised of words from the 1000 most common words found in third-grade schoolbooks,^22^ although the sentences are not necessarily on a third-grade level. We chose to determine eligibility based on reading ability rather than grade in school, as our clinical experience suggests that a portion of children in less than third grade can meaningfully participate in MNREAD testing; therefore, first and second graders were also included if they had the reading ability to do so.

### Procedures

Parents provided information about birth history, ocular diagnosis, medical conditions, medications, and school (grade, accommodations, and services). Visual acuity was measured using the Emmes EVA Visual Acuity Tester (Jaeb Center for Health Research, Tampa, FL) at a 3-m test distance using the standard protocol^23^ after best correction. Right eye (OD), left eye (OS), and binocular (OU) acuity was measured. EVA scores were converted to logMAR with the following formula: 1.7–[(0.02)(letter score)]. Visual field testing was not part of the testing protocol, but visual field results from the clinical record were reviewed.

MNREAD testing was conducted binocularly using the patient's habitual correction for reading and MNREAD test 1 and 2 (Precision Vision, Inc., LaSalle, IL). Two reading conditions were used for participants with VI: fixed 20-cm distance or preferred distance. For preferred distance testing, participants with VI were permitted to get closer to the card as needed in order to read the print. Participants with VI were randomized to use either chart 1 or chart 2 at a fixed 20-cm distance; the remaining chart was tested at their preferred distances. Only data for the 20-cm fixed test distance are presented here. The order of testing was also randomized. Participants with normal vision read both charts 1 and 2 at the fixed 20-cm distance, but the order of presentation was randomized. The card was placed on a reading stand, and the 20-cm testing distance was maintained through the use of strings attached to the card. Sentences were covered and revealed one at a time during testing. Participants were instructed to read each sentence as quickly as possible without making mistakes, and they were instructed not to fix any mistakes but instead to finish reading the sentence. A second examiner timed the passage reading and recorded results to the nearest 0.1 second, as well as errors.

The following parameters were determined for the MNREAD test: maximum oral reading rate, critical print size, and reading acuity, as recommended in the test instructions, accounting for errors. The MRR was determined as the mean of the three fastest reading rates. The smallest print size that could be read at 90% of the maximum reading rate was designated the CPS, and RA was the smallest print read adjusted for errors. The MNREAD cards are labeled for a 40-cm reading distance and were adjusted by 0.3 logMAR because the test was done at 20 cm.

The BRI is a test used in the educational setting and was chosen to assess the validity of the MNREAD test because it is straightforward to administer and is not used in the school districts where we recruited participants. The test offers graded word passages through eighth grade. In this test, students were asked to choose between a regular print version (ranging from an *x*-height of 2.25 mm for third-grade lists/passages to 1.75 mm for eighth-grade passages) and a large print version (6–7 mm *x*-height for all passages). All passages are 100 actual words in length; however, words read per minute were calculated using standard-length words (a standard-length word is six characters long) as recommended by Legge,^1^ rather than the actual word count. The passages ranged from 84.8 to 98.1 standard words (mean, 90.9 words). Participants were permitted to hold the print at their preferred working distance, as would be done in the school setting. The MNREAD CPS was not known prior to BRI testing, as participants completed the BRI first to determine eligibility for MNREAD testing. The independent reading level was determined per the instruction manual. The reading rates determined for independent passage reading levels were used for analyses. Participants were asked to read the passages out loud as quickly as possible. MNREAD and BRI testing was repeated 1 to 3 weeks later, according to the same randomization scheme and administration and scoring protocols used at the initial visit.

### Data Analysis

Data were analyzed using SAS 9.4 (SAS Institute, Inc., Cary, NC). Figures were created with Prism 8 (GraphPad Software, San Diego, CA). We used *t*-tests to detect differences between children with and without vision impairment for continuous data (pooled *t*-tests for variables with equal variance and Satterthwaite *t*-tests for those with unequal variances). Categorical data were compared using χ^2^ tests unless more than 20% of the cell frequencies were less than 5, in which case a two-sided Fisher's exact test was used instead. Test–retest repeatability was measured using intraclass correlations (ICCs). Coefficients of repeatability (CRs) and 95% confidence intervals (CIs) were calculated. Reading speeds were analyzed as the logarithm of words per minute (logWPM). Bland–Altman plots were used to graphically evaluate differences between test and retest values. Linear regression was used to determine the relationship between grade and reading speed. Statistical significance was set at *P* < 0.05, two-tailed.

## Results

Letters were sent to 99 parents of children with VI in grades 1 through 12 who were patients of the first author and who did not have a history of developmental delay or cognitive impairment. Of those, 78 agreed to participate. Forty-four children with normal vision were enrolled, one of whom was screened out due to a diagnosis of dyslexia. Sixteen children with VI (eight of whom were in first or second grade) and four children with normal vision were excluded from analysis due to an inability to read third-grade word lists on the BRI at an independent level and/or a total standard score on the Slosson Intelligence Test of 85 or less. No children in first grade were included in the analysis; however, three of six second-graders with VI met the inclusion criteria.

There were 62 children with vision impairment and 40 children with normal vision who met the entry criteria and were included in these analyses. The children with VI were similar to children with normal vision with respect to age, gender, race, intelligence, and number of adults in the household (Table 1). However, children with VI were more likely to live in a household with income of less than $30,000 per year. Table 2 details the visual characteristics of the participants. Albinism was the most frequent cause of VI, followed by retinal dystrophies or degenerations and optic atrophy. Two-thirds of the VI group had nystagmus. The mean best-corrected binocular visual acuity was 0.6 ± 0.2 logMAR (20/80) for children with VI and –0.13 ± 0.04 (20/15) for children with normal sight. Children with normal sight also performed better on contrast sensitivity testing. Only one participant, with Stargardt macular degeneration, had a detectable central scotoma.

**Table 1.** Demographic Characteristics of Study Population

	Participants with	Participants with	
	Vision Impairment (*n* = 62)	Normal Vision (*n* = 40)	*P* (*df*)^a^
Age (y), mean (SD)	13.3 (3.0)	13.3 (2.6)	0.99 (100)
Gender, *n* (% male)	40 (64.5)	20 (50.0)	0.15 (1)
Race, *n* (%)			0.7
White	42 (67.7)	30 (75)	
Black	15 (24.2)	8 (20.0)	
Other	5 (8.1)	2 (5.0)	
Premature birth, *n* (%)	7 (11.3)	5 (12.5)	1.0
Screening Intelligence Total Standard Score, mean (SD)	107.9 (14.5)	106.3 (12.4)	0.6 (100)
School setting, *n* (%)			0.01^*^
Public	38 (61.3)	30 (75.0)	
Private	10 (16.1)	2 (5.0)	
Homeschool	5 (8.1)	8 (20.0)	
School for the blind	9 (14.5)	0 (0.0)	
Receives special services at school, *n* (%)			<0.01^*^
No service or accommodations	3 (4.8)	40 (100.0)	
Accommodations but no direct services	30 (48.4)	0 (0.0)	
Direct services of non-vision specialist	3 (4.8)	0 (0.0)	
Direct services of teacher of the visually impaired	8 (12.9)	0 (0.0)	
Direct services of teacher of the visually impaired and mobility specialist	7 (11.3)	0 (0.0)	
Extensive services at school for the blind	9 (14.5)	0 (0.0)	
Orientation and mobility services only	2 (3.2)	0 (0.0)	
Uses an electronic video magnifier at home, *n* (%)	24 (38.7)	0 (0.00)	<0.01^*^ (1)
Family income, *n* (%)			0.01^*^ (2)
Less than $29,999	14 (22.6)	3 (7.5)	
$30,000 or more	46 (74.2)	29 (72.5)	
Declined to answer	2 (3.2)	8 (20.0)	
Number of adults in household, *n* (%)			0.4 (1)
One	10 (16.1)	4 (10.0)	
Two or more	52 (83.9)	36 (90.0)	
Number of children in household, mean (SD)	2.1 (0.9)	2.7 (1.3)	0.008^*^ (65.4)

Comparisons used *t*-tests for continuous variables (pooled for equal variances and Satterthwaite for unequal variances) and χ^2^ tests for categorical variables or Fisher's exact test if >20% of cell frequencies were <5.

a If degrees of freedom are not indicated, a Fisher's exact test was used.

*

**Table 2.** Visual Characteristics of Study Population

	Participants	Participants	
	with Vision	with Normal	
	Impairment (*n* = 62)	Vision (*n* = 40)	*P* (*df*)^a^
Ocular diagnosis, *n* (%)			<0.001^*^
Achromatopsia, cone dystrophy	7 (11.3)	0 (0.0)	
Albinism, congenital nystagmus	27 (43.6)	0 (0.0)	
Optic atrophy	8 (12.9)	0 (0.0)	
Optic nerve hypoplasia	4 (6.5)	0 (0.0)	
Other	6 (9.7)	0 (0.0)	
Retinal degeneration/dystrophy	8 (12.9)	0 (0.0)	
Retinopathy of prematurity	2 (3.2)	0 (0.0)	
None (control)	0 (0.0)	40 (100.0)	
Best-corrected distance visual acuity (logMAR), mean (SD)			
OD	0.7 (0.2)	–0.08 (0.05)	<0.001^*^ (67.8)
OS	0.7 (0.3)	–0.09 (0.05)	<0.001^*^ (64.0)
OU	0.6 (0.2)	–0.12 (0.04)	<0.001^*^ (67.0)
Reading acuity OU (logMAR), mean (SD)	0.57 (0.23)	–0.15 (0.10)	<0.001^*^ (67.0)
Nystagmus, *n* (%)	41 (67.2)	0 (0.00)	<0.001^*^ (1)
Mars Contrast Sensitivity, mean (SD)	1.6 (0.2)	1.8 (0.05)	<0.001^*^ (70.8)

Comparisons used *t*-tests for continuous variables (pooled for equal variances and Satterthwaite for unequal variances) and χ^2^ tests for categorical variables or Fisher's exact test if >20% of cell frequencies were <5.

a If degrees of freedom are not indicated, a Fisher's exact test was used.

*

**Figure 1.**
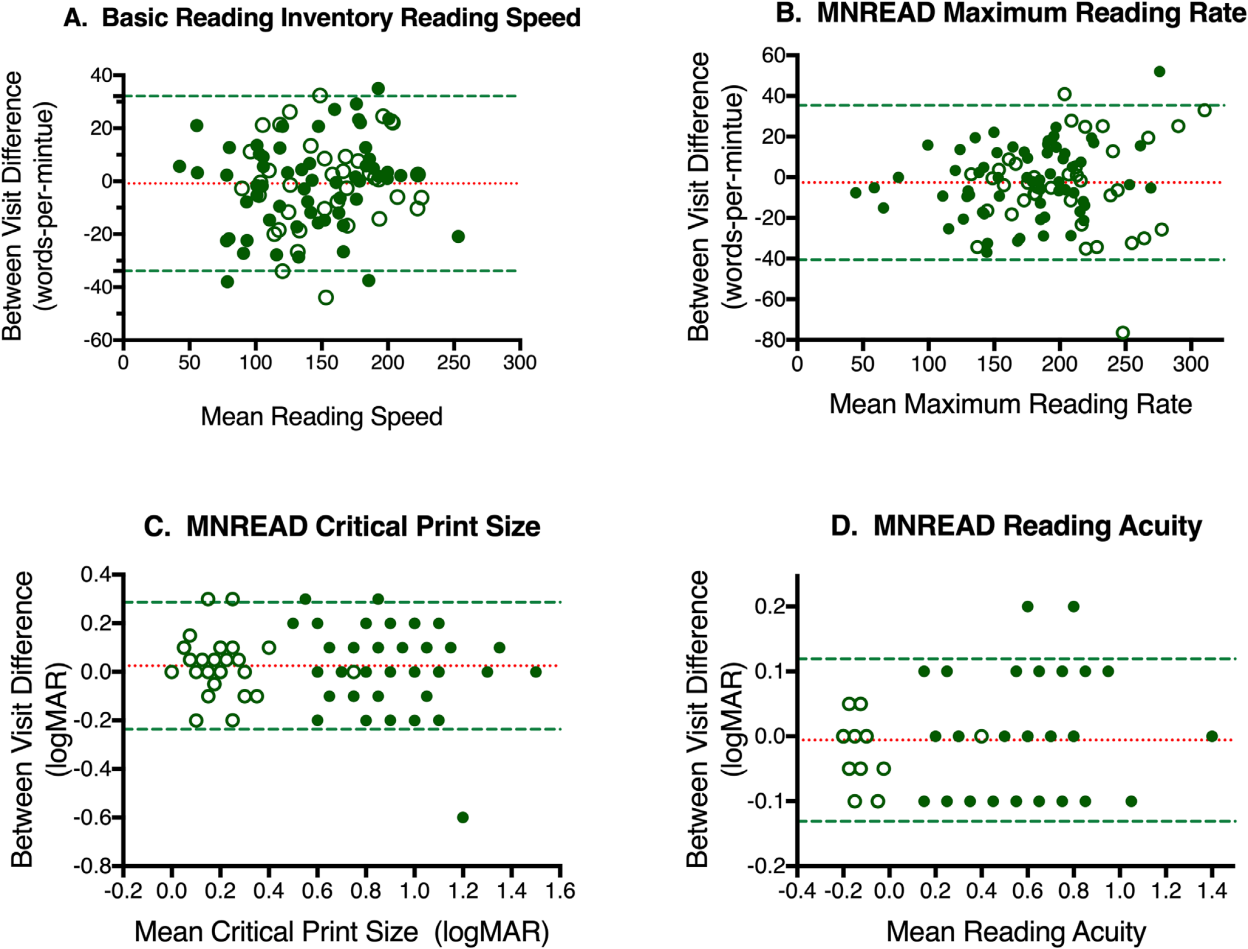
Difference versus mean plots (Bland–Altman) comparing results from visits 1 and 2. *Open circles* denote participants with normal vision, and *closed circles* denote participants with VI. *Red dotted lines* indicate the mean difference between the two measures. *Green dashed lines* indicate ±1.96 SD.

Difference versus mean plots (Bland–Altman) are presented for RA, CPS, MRR, and the BRI reading rate for the entire sample in order to illustrate test–retest relationships (Fig. 1). None of the slopes was significantly different than zero when analyzed for each vision status independently or for the combined data, suggesting a lack of systematic bias among the measurements.

There is very strong agreement overall between test and retest values for MNREAD RA, CPS, and MRR, as well as the BRI reading rate, with most correlations being >0.9, the exception being CPS, for which the correlations were lower but still strong (Table 3). The CRs can be found in Table 3. For MNREAD testing, the CRs for reading acuity and CPS were greater for children with VI; however, the CRs for BRI reading rates were lower for children with VI. The CRs for reading rates on the MNREAD were nearly the same for both groups.

**Table 3.** Coefficients of Repeatability and Intraclass Correlations for MNREAD Parameters and BRI Reading Rate for Test–Retest

		MNREAD Reading Acuity	MNREAD Critical Print Size	MNREAD Maximum Reading Rate	BRI Reading Rate
Overall	CR	0.13 (0.11–0.14)	0.26 (0.24–0.29)	0.21 (0.19–0.23)	0.26 (0.24–0.29)
	ICC	0.99	0.95	0.95	0.92
Vision impairment	CR	0.15 (0.13–0.17)	0.3 (0.26–0.34)	0.21 (0.18–0.24)	0.28 (0.24–0.32)
	ICC	0.95	0.77	0.96	0.92
Control	CR	0.07 (0.06–0.08)	0.2 (0.17–0.23)	0.20 (0.17–0.24)	0.23 (0.19–0.27)
	ICC	0.93	0.68	0.89	0.89

**Figure 2.**
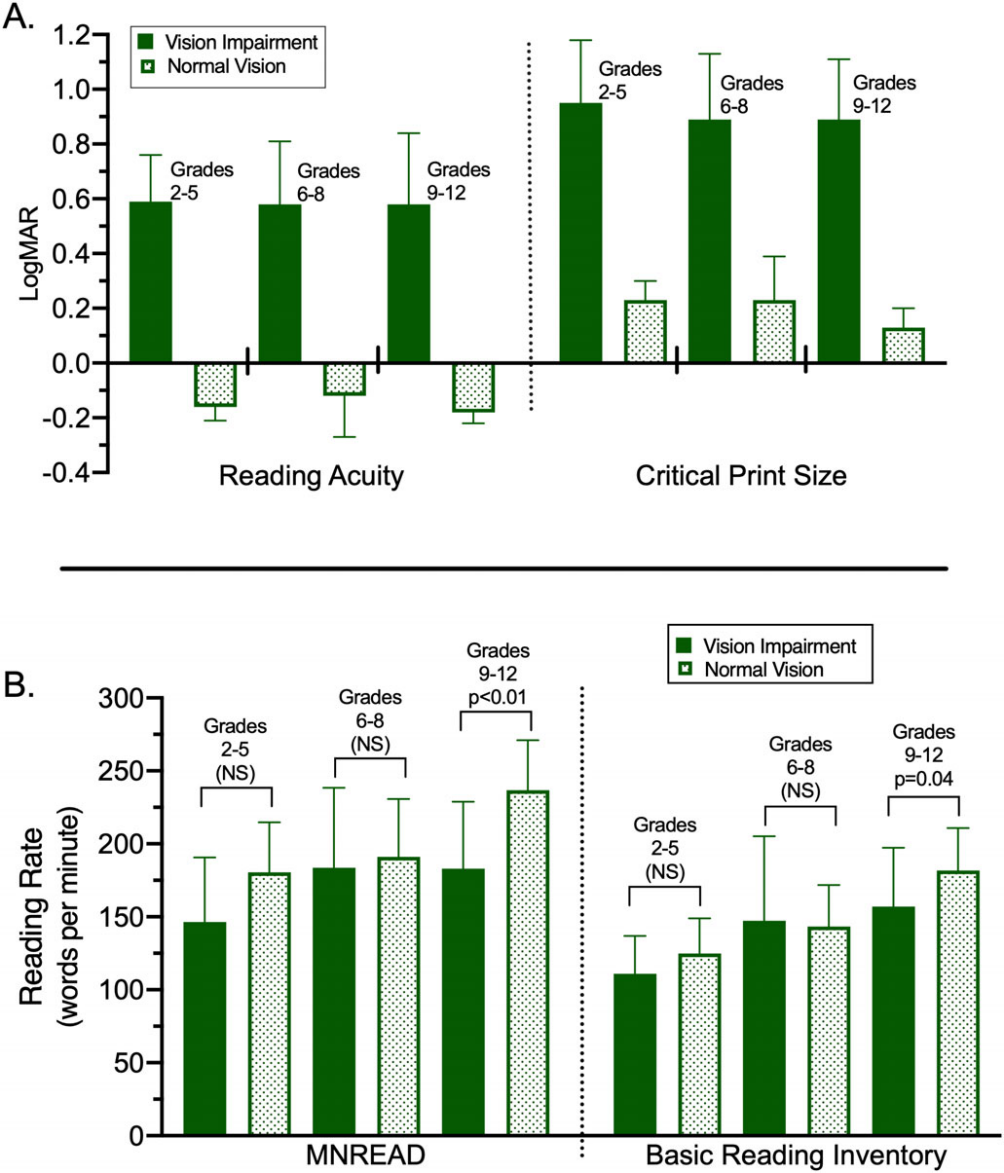
Comparison of (A) MNREAD RA and CPS and (B) MNREAD MRRs and BRI reading rates between children with and without vision impairment by grade levels. Average values for each participant over the two visits were compared using *t*-tests (pooled for equal variances and Satterthwaite for unequal variances). All differences in A were statistically significant. Significance is indicated on the graph in B. Error bars indicate standard deviation. NS, not significant.

Comparing children with VI to those without VI, there were significant between-group differences in RA and CPS, as the groups were designed to differ in visual ability (Fig. 2). Although children with VI on average read more slowly on both the MNREAD test and the BRI, the difference was only statistically significant for those in grades 9 to 12. As expected, both RA and CPS were significantly different (*P* < 0.001) between participants with and without VI across all grade groups. Within the VI group, there were no differences in either MNREAD or BRI reading rates between those with and without nystagmus (*P* = 0.37 and *P* = 0.33, respectively).

To assess the validity of the MNREAD test, we compared the reading rates from the MNREAD test and the BRI. Although the values were strongly correlated (Pearson's *r* = 0.88), they still differed significantly both overall and when grouped by vision status. The MNREAD test yielded faster reading speeds than the BRI. Reading speeds on the MNREAD were 31.4 ± 21.5 wpm faster for children with VI and 52.3 ±19.5 wpm faster for children with normal vision than reading speeds on the BRI.

**Figure 3.**
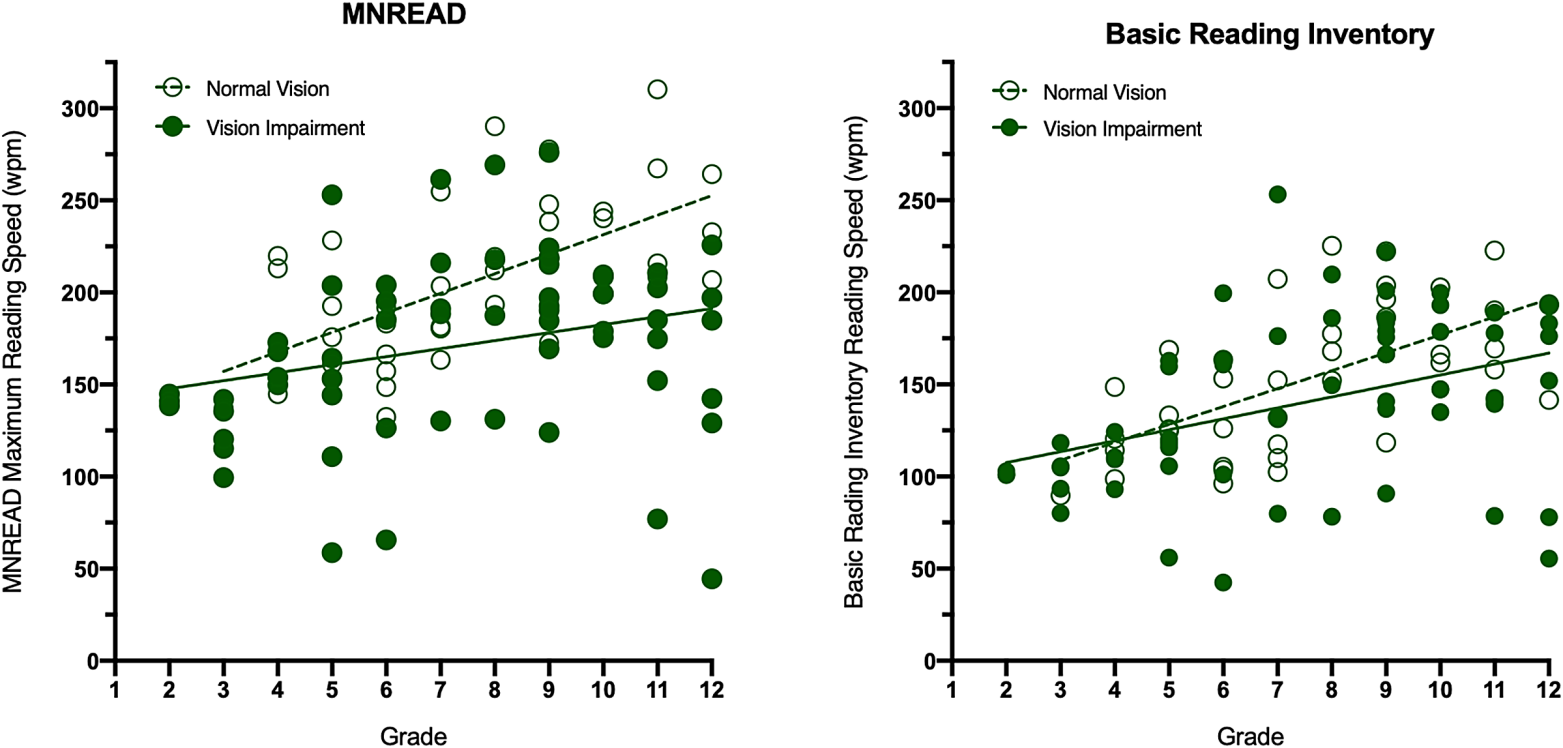
Reading rate in words per minute by grade for the MNREAD test (*left*) and BRI (*right*).

When looking at reading speed by grade level, among children with vision impairment, reading speed increased, on average, 4.4 wpm (95% CI, 0.3–8.4) for the MNREAD test and 5.9 wpm (95% CI, 2.4–9.5) for the BRI each year. Children without vision impairment increased their reading speed 10.6 wpm (95% CI, 6.2–15.0) for the MNREAD test and 9.7 wpm (95% CI, 5.8–13.7) for the BRI each year. (Fig. 3) There was no difference between the slopes of the lines generated for children with VI or normal sight by linear regression for either test. For the MNREAD MRR, *F* = 3.668, degrees of freedom numerator (*DFn*) = 1, degrees of freedom denominator (*DFd*) = 98, and *P* = 0.0584; for the BRI MRR, *F* = 1.695, *DFn* = 1, *DFd* = 98, *P* = 0.1960.

**Figure 4.**
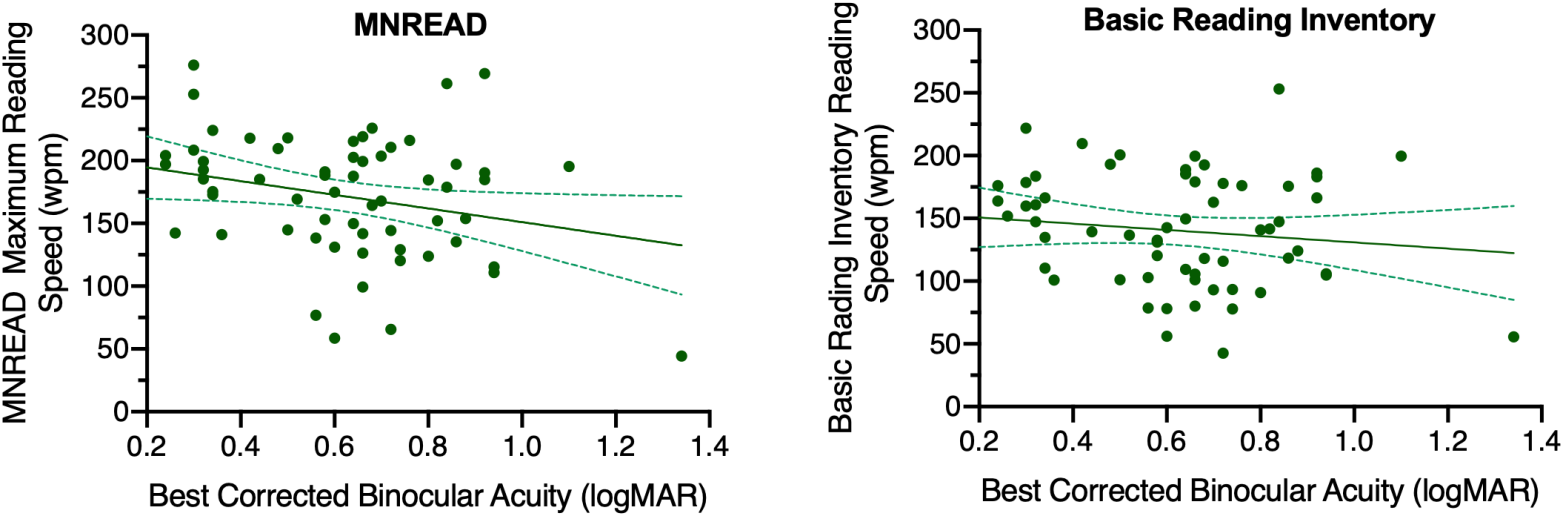
Relationship of reading rate for the MNREAD test (*left*) and BRI (*right*) to visual acuity for participants with vision impairment. Dotted lines indicate 95% CIs.

The association between best-corrected binocular visual acuity and reading rate for both the MNREAD test and the BRI (reading rate for each test averaged over the two visits) was investigated using univariate regression (Fig. 4). There was a significant association between poorer visual acuity and lower reading rates on the MNREAD test (Pearson's *r* = –0.26, *P* = 0.04) but not on the BRI (Pearson's *r* = –0.13, *P* = 0.3).

## Discussion

The MNREAD test showed good test–retest reliability for children with VI and for those with normal vision. CPS varied the most from visit 1 to visit 2, with the majority of results falling within 0.2 logMAR (two lines of print size). Reading speed is highly variable from person to person; however, the intraclass correlations were excellent for both reading tests. These results are similar to those of Virgili et al.,^18^ who examined the use of the MNREAD test in Italian children with normal vision, and Subramanian and Pardhan,^24^ who examined their use in adults with low vision.

The CRs in our study show between-visit differences that are similar to the findings of other studies. Italian children in grades 3 to 8 with normal near vision were evaluated using two different versions of the MNREAD chart on the same day.^18^ That study reported CRs of ±0.14 logMAR for RA, ±0.19 logMAR for CPS, and ±0.08 logWPM for MRR. Repeatability was somewhat better among adults with normal vision: ±0.05 logMAR for RA, ±0.1 logMAR for CPS, and 8.5 WPM for MRR.^25^ The only report of repeatability of MNREAD indices among persons with VI studied adults, most of whom had age-related macular degeneration. That study reported CRs of ±0.1 logMAR for RA, ±0.20 logMAR for CPS, and ±0.10 logWPM for MRR.^24^ There were no significant differences in repeatability when dividing the participants into two groups: (1) 0.5 logMAR or better, and (2) worse than 0.5 logMAR but better than 1.3 logMAR. The average RA and CPS values for children with VI in our study were similar to those for the adults with VI in Subramanian and Pardhan's study.^24^ Consistent with these studies, our children with low vision had more variability between tests than the children in our study with normal vision. It is not surprising that CPS is the most variable parameter, as it is measured in the largest steps (0.1 logMAR as compared to 0.01 logMAR for RA). Among children with VI, the CPS varied by ±0.3 logMAR, which represents a doubling of the visual angle. These findings provide useful information about how much change in reading performance must be experienced to indicate that a true clinical change has taken place. These types of measures are of increasing importance as new therapies are being developed to target inherited retinal conditions that are common among children with VI.

The MNREAD reading speeds in our study were similar to those of Calabrèse et al.,^26^ who found that at age 8 children with normal vision read on average 137 wpm, a rate that increased by 8.13 wpm/y until the children were age 16, when their reading speeds plateaued around 202 wpm. The children without VI in that study exceeded the reading speeds in the cohort and increased their reading speeds by more words per year; however, although younger children read similarly to their normally sighted peers, the older children with VI did not reach 202 wpm. This is not surprising, as reading speeds in our VI group increased annually by half of that of Calabrèse's cohort. The students with VI in our study did not reach a plateau in reading speed, unlike the study of Corn et al.,^9^ who found that reading speeds plateaued under 100 wpm after sixth grade among readers with VI.

The MNREAD maximum reading speed is an average of the three fastest reading speeds on the test and is much faster than the reading speeds for the BRI. The BRI is a paragraph reading test, so the reading rates would not be expected to be identical between the two tests; however, the strong correlation between the two tests supports the validity of the MNREAD test. It is well known that the type of reading being done impacts reading speed. Several possibilities exist as to why reading speeds have been found to be greater on the MNREAD test. First, the MNREAD test are short enough that the reader does not need to take a breath during reading of the sentence and they are instructed to read as quickly and accurately as possible. Second, readers may become fatigued over the course of reading a paragraph; adult readers with glaucoma have been shown to read more slowly on longer passages.^27^ Third, readers may read more slowly as they try to comprehend the paragraph (although no instructions regarding comprehension were given and no comprehension questions were asked). Fourth, the passages being read on the BRI were at the child's independent reading level up to the eighth-grade level which is the maximum for the test, so the complexity of the passage may have been greater. Finally, it is possible that some children were reading print on the BRI that was smaller than their CPS.

The CPS among children with VI had a CR that was large for the MNREAD test (0.3 logMAR). Given the variability even within the same test, it is possible that longer tests, such as the BRI have a different CPS; however, findings were similar for the normally sighted control group for whom all passages would be greater than their CPS. This makes it unlikely that the print sizes on the BRI were the primary reason for slower reading rates.

Two-thirds of the participants with VI in this study had nystagmus, and one might attribute slower reading speeds among the VI group to nystagmus; however, we found similar reading rates among children with and without nystagmus. Woo and Bedell^28^ showed that people with nystagmus are reading during non-foveating periods. This was further supported by Dysli and Abegg,^29^ who found that, although latency to initiating reading of an eight-letter word was longer, first fixation duration was shorter and the number of fixations were greater among participants with nystagmus. Text reading speeds were the same as healthy controls. Wang and Dell'Osso^30^ described the concept of children with nystagmus being “slow to see.” They found that the oculomotor system utilizes foveating and braking saccades to adapt to the underlying nystagmus and that the foveation periods following foveating saccades facilitate how well the person sees. They proposed that these periods have a negative effect on how quickly they see, making target acquisition time an additional factor in visual function.

The concept of being “slow to see” could explain why there are greater percent differences in reading speed between readers with VI and those with normal sight on the MNREAD test versus the BRI. Although, in general, VI readers do read faster on the MNREAD than the BRI, they may be “slow to see” and therefore take longer on a shorter passage than their normally sighted counterparts, who are able to begin as soon as the sentence is revealed. These shorter sentences likely reflect differences in their ability to perceive the stimulus, whereas the longer passages would be more dependent on other skills such as grammar and linguistics.^29^^,^^30^ Despite the differences between children with and without VI, in our study the reading speeds in the group of children with VI were faster than those reported in the literature.

A strength of this study is that the participants were screened to be sure that they were not cognitively impaired and that they were able to read at an independent reading level of at least grade 3. Some causes of pediatric low vision such as septo-optic dysplasia^31^ or retinopathy of prematurity^32^ are associated with other disabilities, and having vision impairment does not protect a child with low vision from having a reading disability. By restricting enrollment to those without cognitive or reading deficits, we are able to measure reading speed without those potential confounders. Additionally, 62 children with VI is a large sample size given the prevalence of pediatric VI in the US population.

## Conclusions

The MNREAD test show good test–retest reliability and criterion validity and are useful in the evaluation of reading in children with VI, but they should be interpreted with caution, as they may overestimate reading ability for longer passages. As the CPS is the most variable parameter across visits, it may be necessary to determine the CPS on more than one occasion before using this information to recommend print sizes for educational purposes.
